# Insights in ischemia/reperfusion injury and cardioprotection: neglected and emerging pathways and therapeutic targets for a personalized therapy

**DOI:** 10.1007/s00395-026-01167-8

**Published:** 2026-03-13

**Authors:** Pasquale Pagliaro, C. Penna, S. Femminò, F. G. P. Welt

**Affiliations:** 1 Department of Clinical and Biological Sciences, University of Torino, Turin, Italy; 2 Division of Cardiovascular Medicine, Department of Medicine, University of Utah Hospital, Salt Lake City, UT USA

**Keywords:** Cardioprotective pathways, Cardioprotection, Ischemia/reperfusion injury, Emerging targets

## Abstract

Despite extensive preclinical research identifying molecular targets and cardioprotective strategies, translation into effective clinical therapies remains challenging. Cardioprotection aims to mitigate ischemia/reperfusion injury (IRI) by modulating molecular pathways, such as the Reperfusion Injury Salvage Kinase (RISK) and Survivor Activating Factor Enhancement (SAFE) pathways, as well as autophagy, inflammation, and regulated cell death, to preserve myocardial function. However, a major limitation lies in the robustness of preclinical evidence. Many experimental studies rely on simplified models that fail to reproduce the complexity of human cardiac pathophysiology, resulting in inconsistent and poorly reproducible cardioprotective effects. It is likely that RISK-SAFE pathways represent an oversimplified framework. Moreover, most experimental approaches are cardiomyocyte-centered, overlooking the critical role of the vessels in IRI. Clinical translation is further compromised by patient-related factors, including comorbidities (e.g., diabetes, hypertension), concomitant medications, and heterogeneity in reperfusion protocols, all of which attenuate cardioprotective efficacy. Additional variables, such as timing of intervention and species differences, further contribute to translational failure. Emerging approaches include pharmacological therapies (e.g., SGLT2 inhibitors, PARP inhibitors, necroptosis and ferroptosis blockers, NLRP3-targeting compounds), cell- and organelle-based strategies (e.g., mitochondrial transplantation, extracellular vesicles, non-coding RNAs), and mechanical/device-based interventions (e.g., left ventricular unloading, ischemic conditioning, controlled reperfusion, selective intracoronary hypothermia). Future research should emphasize multi-target interventions, optimized timing and delivery, and advanced tools, such as nanocarriers, gene therapy, computational modeling, and adaptive clinical trials. Strengthening the robustness of preclinical models, including human ex vivo cardiac systems, remains essential to bridge the translational gap and improve the clinical success of cardioprotective therapies.

## Introduction

**Open problem:** Despite major advances in systems of care for patients with acute myocardial infarction (AMI) and prompt reperfusion, AMI remains a leading cause of morbidity and mortality worldwide [222]. Decades of research have clearly demonstrated that a substantial contributor to adverse outcomes in AMI relates to ischemia/reperfusion injury (IRI) [5, 82]. A major breakthrough in the treatment of IRI occurred in 1986 with the discovery of the beneficial effects of ischemic preconditioning (IPC) [190]. While not necessarily a clinically applicable strategy, this discovery raised the intriguing possibility that with a better understanding of the molecular pathways responsible for this injury, treatments could be developed to further reduce morbidity and mortality in AMI. Since then, nearly 53,500 manuscripts indexed in PubMed have addressed the topic of “ischemia–reperfusion” with approximately 31,130 specifically focusing on cardioprotection (PubMed, 2025 November 10).

**General explanation:** Cardioprotection—defined as the enhancement of myocardial resistance to ischemia/reperfusion stress—has long been a critical goal in both basic and clinical cardiovascular research. The goal is to reduce the IRI and preserve cardiac function. This includes strategies, such as IPC, which, as said above, uses brief ischemic episodes to reduce the impact of more severe ischemia [22, 71, 246].

Since its 1986 description, the concept of ischemic conditioning has grown from the original/local IPC—brief, nonlethal ischemia applied before a major insult that produces an immediate cardioprotective “first window” of protection—into a temporal and spatial spectrum of protective strategies [68, 106, 161, 244].

Crucially, a late (delayed) window of protection (also called delayed/late ischemic preconditioning or the “second window of protection”) was later recognized: it appears ~ 24–72 h after the conditioning stimulus and is mediated in large part by gene transcription and new protein synthesis (e.g., inducible nitric oxide synthase, heat-shock proteins and antioxidant enzymes), producing longer-lasting cellular adaptations [15, 21, 241].

Additional forms include ischemic postconditioning (PostC: interrupting flow at early reperfusion) [172, 180, 252], remote ischemic conditioning (RIC: conditioning ischemia performed in organ distant from the target organ) [67, 75, 183] and its timing variants (remote pre-, per- and post-conditioning), as well as repetitive/chronic conditioning (repeated RIC over days–weeks) aimed at sustained protection.

Altogether, these immediate and late, local and remote, protective protocols form the continuum of “ischemic conditioning” still under active translational and clinical investigation [83, 173].

**Limits of studies:** Table 1 provides an overview of how different conditioning strategies have evolved from experimental discovery to variable clinical application, emphasizing areas of reproducible efficacy and controversies. Recently, efforts are on the way to overcome these controversies. For instance, a couple of studies within the IMPACT initiative, using small animal and pig models, investigate the cardioprotective effects of ischemic preconditioning in acute myocardial infarction. Together, they aim to enhance the translational relevance of preclinical research and bridge the gap toward effective clinical therapies [72, 106].

**Table 1:** Comparative overview of major ischemic conditioning paradigms and their translational evidence

Conditioning Paradigms	Original study (first report)	Recent and comprehensive reviews	Type of protection *(cardiac/cerebral/other)*	Key translational study	Notes/observations (interpretation, controversy, limitations)
Ischemic preconditioning (IPC)	[161]	[68]	Cardiac	[244]	Foundational concept; translation limited by protocol heterogeneity and AMI unpredictability
Remote ischemic conditioning (RIC)	[183]	[75]	Cardiac/cerebral/renal	[67]	Outcome variability due to timing and patient comorbidities
Pharmacological preconditioning (adenosine, etc.)	[129]	[52]	Cardiac	[198]	Species differences and pharmacological limitations
Ischemic postconditioning (PostC)	[252]	[172]	Cardiac	[180]	Human trials failed to reproduce experimental benefit
Second window of protection (SWOP)	[15]	[21]	Cardiac	[241]	Limited feasibility in acute/emergency conditions

**Limits in translations:** While several strategies have shown promise in preclinical settings, their translation into effective clinical therapies has been largely disappointing [78, 97]. Besides the obvious issue that one cannot apply ischemic pre-conditioning prior to an unexpected AMI in the field, the “bench-to-bedside” translation failure of per- and post-conditioning can be attributed to several factors, including the limitations of preclinical models in replicating the complexity of human diseases and the potential for unforeseen interactions in the human body [72, 77, 106]. This gap underscores the need to revisit fundamental mechanisms of injury and protection, with renewed attention to the complexity of molecular signaling, cellular interactions, and the timing of therapeutic interventions.

**Novel mechanisms to be considered:** Beyond the classical ischemic conditioning paradigm, recent research has identified several novel molecular and cellular mechanisms that may modulate myocardial injury and protection, including immune and inflammatory pathways, mitochondrial dynamics, and metabolic reprogramming [8, 24, 33].

**Aim:** Recent reviews have proposed several pharmacological and non-pharmacological approaches to limit IRI [20, 79, 102, 156, 210, 212, 230]. In the present review, we focus on some neglected and recently characterized mechanisms of myocardial injury and highlight emerging molecular and cellular targets that warrant further investigation, both in preclinical models and in clinical settings.

## Mechanistic basis of ischemia–reperfusion injury and classical and novel molecular pathways of cardioprotection

### Main mechanisms of myocardial IRI

IRI results primarily from a complex interplay of metabolic, ionic, and oxidative disturbances that occur during ischemia and upon restoration of blood flow [79].

Myocardial ischemia causes intracellular acidosis and calcium overload, impairing the mechanical and electrical function of cardiac cells and promoting arrhythmias [79]. The activation of the Na^+^/H^+^ exchanger (NHE), as an attempt to correct acidosis, leads to an accumulation of intracellular Na^+^, which in turn inhibits Ca^2+^ extrusion through the Na^+^/Ca^2+^ exchanger (NCX), thereby contributing to calcium overload [163]. During ischemia, ATP-sensitive potassium channels open, shortening the action potential and reducing Ca^2+^ influx—an adaptive mechanism limiting energy consumption. However, chronic hypoxia, as observed in heart failure, may prolong the action potential due to downregulation of transient K^+^ channels, predisposing to calcium overload and arrhythmias. Calcium overload within the sarcoplasmic reticulum (SR) can trigger spontaneous diastolic Ca^2+^ releases through ryanodine receptors (RyR2), generating delayed afterdepolarizations (DADs) and potentially causing arrhythmias [53].

Reperfusion, although essential, can exacerbate injury through a rapid rise in intracellular Na^+^ and oxidative stress, damaging membranes and ion channels. During reperfusion, mitochondria may open the mitochondrial permeability transition pores (mPTP), leading to membrane depolarization, ATP depletion, cytochrome c release, and cell death [247]. Finally, connexin 43 (Cx43), a protein located on both the plasma and mitochondrial membranes and essential for intercellular communication, becomes altered during ischemia/reperfusion, contributing to the electrical and metabolic dysfunction of the myocardium [197]. During early reperfusion, a burst of reactive oxygen species (ROS) arises primarily from mitochondrial complexes I and III, NADPH oxidases, and xanthine oxidase, causing oxidative damage to lipids, proteins, and nucleic acids [259]. Mitochondria are both a major source and a primary target of oxidative stress; excessive ROS generation contributes to calcium overload, mPTP opening, and subsequent cell death through necrosis, apoptosis, and regulated necroptosis. Recent work has refined the understanding of these redox-dependent mechanisms, identifying specific ROS sources and their spatial compartmentalization within cardiomyocytes as determinants of injury severity [80].

Post-ischemic contractile dysfunction, or “stunning,” is associated with troponin I degradation and reduced Ca^2+^ sensitivity of myofilaments. In addition to these cardiomyocyte alterations, effects on the coronary vasculature, including the no-reflow phenomenon, play a crucial role in determining the extent of myocardial IRI (see below).

IRI should not be regarded merely as an experimental or pathophysiological entity; rather, it represents a clinically relevant process that contributes to measurable major adverse cardiovascular events (MACE) and significantly impacts patient outcomes. In patients with acute myocardial infarction undergoing reperfusion therapy, the extent of IRI is commonly assessed by markers, such as infarct size, microvascular obstruction, intramyocardial hemorrhage, or impaired myocardial salvage on cardiac magnetic resonance imaging [116, 221]. These parameters have been consistently associated with an increased incidence of MACE, including cardiovascular death, recurrent myocardial infarction, heart failure hospitalization, and unplanned revascularization during follow-up [73, 237]. Collectively, these data underscore the clinical significance of IRI and support ongoing efforts to identify therapeutic strategies aimed at limiting these damages and events in order to improve long-term cardiovascular outcomes.

### RISK and SAFE and NO/cGMP pathways

Cardioprotective signaling pathways converge on mitochondrial targets to limit injury and preserve cellular integrity. Over the past three decades, two major prosurvival signaling pathways have dominated the field of cardioprotection: the Reperfusion Injury Salvage Kinase (RISK) pathway and the Survivor Activating Factor Enhancement (SAFE) pathway, which cross-talk with the nitric oxide (NO) cyclic guanosine monophosphate (cGMP) pathway. Although these pathways are well-characterized and neither novel nor overlooked, a concise overview is essential to contextualize the additional mechanisms discussed in this review.

The RISK pathway encompasses phosphatidylinositol-3-kinase (PI3K)—Akt and extracellular signal-regulated kinase (ERK1/2) signaling cascades, while the SAFE pathway primarily involves janus kinase (JAK)—signal transducer and activator of transcription 3 (STAT3) signaling. The RISK pathway, often associated with cardioprotection during reperfusion, involves the activation of both PI3K—protein kinase B (Akt) and ERK1/2 signaling pathways; both converge on glycogen synthase kinase 3β (GSK3 β). However, the exact role of each pathway in RISK may vary depending on the context [35, 245]. Consistent with current knowledge of STAT3 function within the SAFE pathway, the ambivalent role of STAT3 in myocardial IRI is evident, exhibiting a protective effect when activated in the early phases of reperfusion and a detrimental effect in the later stages [37, 104].

Both RISK and SAFE converge on the inhibition of mPTP opening, a critical event in the execution of cell death after reperfusion [41, 160]. Despite preclinical success, the modulation of RISK and SAFE alone has proven insufficient in clinical trials, suggesting a more complex network of interacting mechanisms [212]. For instance, in RIC the spleen acts as a relay organ, releasing cardioprotective humoral factors upon vagal activation in rats, pigs, and humans [123].

RISK and SAFE pathways are integrated by the cGMP signaling pathway. This pathway plays a crucial role in cardiovascular physiology by regulating vascular tone, cardiac contractility, cell growth, and apoptosis. In the heart, cGMP is primarily synthesized by two types of guanylate-cyclases: *soluble guanylate cyclase* (sGC), which is activated by NO, and *particulate guanylate cyclase* (pGC), stimulated by natriuretic peptides. cGMP acts as a second messenger by activating protein kinases (PKG) that modulate several cellular processes, including the reduction of oxidative stress, inhibition of pro-inflammatory pathways, regulation of cardiac remodeling, and prevention of programmed cell death. The degradation of cGMP is mainly controlled by *phosphodiesterase type 5* (PDE5), which limits the duration and intensity of cGMP signaling [155]. Dysfunction of the cGMP pathway is linked to various cardiovascular diseases, such as ischemia, cardiac hypertrophy, and heart failure, as it results in increased oxidative stress, inflammation, and fibrosis, ultimately impairing cardiac function [100, 112]. These main pathways are elucidated by several other elements and mechanisms discovered more recently [20, 62, 106, 191], some of which are described below.

### Protein kinases outside the canonical RISK and SAFE pathways

Several kinases have been implicated in myocardial IRI and cardioprotection. Among these, the c-Jun N-terminal kinase (JNK) has attracted considerable attention as a stress-activated MAPK [151]. JNK undergoes rapid phosphorylation during ischemia and reperfusion and has been associated with myocardial injury [199].

Experimental data highlight a complex regulation: ischemic or pharmacological preconditioning increases JNK phosphorylation, whereas PostC reduces it [30, 199]. Remote ischemic conditioning similarly attenuates JNK activation [242]. At the mechanistic level, JNK promotes mitochondrial dysfunction, apoptosis [9, 199], and ROS generation [27]. Of note, JNK has been detected within mitochondria of various cell types and tissues [119, 150, 170], where it localizes to the outer mitochondrial membrane. There, it interacts with the scaffold protein SAB, initiating a signaling cascade that impairs respiration and amplifies ROS production, generating a P-JNK/SAB/ROS feedback loop that culminates in cell death [26, 74, 233].

Mitochondrial JNK has also been linked to maladaptive processes, such as dynamin-related protein 1 (Drp1)-mediated fission and mitochondrial fragmentation [95, 224]. Nonetheless, some studies report that ischemic preconditioning can be associated with increased mitochondrial JNK, suggesting context-dependent protective roles [95].

Overall, JNK signaling represents a non-canonical pathway influencing cardiomyocyte fate during ischemia/reperfusion. Its dual nature—cytoprotective in certain conditioning paradigms but deleterious when chronically activated—underscores the need to better define the conditions that govern mitochondrial JNK import, its binding partners, and its functional outcomes.

Pharmacological inhibition of JNK has been shown to reduce infarct size in several settings [27, 181, 199], although paradoxical aggravation of IRI has also been reported [89]. This apparent duality may depend on ischemia duration and the metabolic state of the myocardium [229]. Other pathways involve Src-Family Kinases, p38 MAPK, PINK1-Mediated Mitophagy, and Hippo-YAP signaling pathway.

#### Src-family kinases (SFKs) as non-canonical modulators of cardioprotection

SFKs represent an additional layer of signaling complexity in myocardial IR. Seven members of this family are expressed in cardiomyocytes, including Fyn, Fgr, Yes, Src, Lyn, Lck, and Blk. Evidence from rabbit models indicates that Src and Lck act downstream of PKCε and may cross-talk with the NO pathway during IPC [179, 220]. Moreover, studies in pigs have shown that the combined inhibition of PKC and tyrosine kinases abolishes the protective effects of IPC, suggesting that Src-related pathways are required for full cardioprotection [218].

Although classically cytoplasmic, several SFKs—including Src, Fyn, Lyn, and Fgr—have been detected in mitochondria across different tissues, including the heart [126, 248]. Their mitochondrial targeting appears to depend on scaffold or docking proteins, such as AKAP-12 [136] and docking protein 4 (Dok-4) [87]. In cardiomyocyte models, hypoxia/reoxygenation reduces mitochondrial Src phosphorylation, an effect counteracted by JNK inhibition or SAB blockade [34, 192]. In rodent hearts, IPC enhances mitochondrial Src Tyr416 phosphorylation during reperfusion, with Src located at complex I of the respiratory chain. Interestingly, phosphorylated Src correlates with reduced complex I activity and decreased ROS generation [61]. Unlike other prosurvival kinases, therefore, mitochondrial Src seems to exert cardioprotection through a controlled inhibition of respiration. However, this effect appears to be tissue-specific, as Src activation enhances oxidative phosphorylation in brain mitochondria [10].

Additional evidence links mitochondrial Src activity to preconditioning stimuli: phosphorylation of the adenine nucleotide translocator 1 (ANT1) has been implicated in isoflurane-induced cardioprotection [51]; morphine exposure increases mitochondrial Src phosphorylation upon reperfusion [69]; and exogenous NO triggers cardioprotection via Src-dependent complex I inhibition and attenuation of oxidative stress [251].

Notably, the role of Src extends beyond mitochondrial regulation. In the context of apelin-APJ signaling, apelin exerts post-conditioning-like protection against IRI through activation of the PI3K-Akt-NO cascade. Experimental studies in isolated rat hearts demonstrated that Src is a critical mediator in this pathway: inhibition of Src prevented apelin-induced reductions in infarct size, attenuation of contracture, and recovery of post-ischemic function, effects that were also sensitive to RISK inhibition. Mechanistically, Src mediates both the transactivation of EGFR via MMP activity and the phosphorylation-dependent inactivation of PTEN, thereby sustaining PI3K/Akt signaling [55]. This dual action highlights Src as a pivotal integrator of GPCR-induced cardioprotective signaling, acting in concert with canonical survival pathways.

Taken together, these findings support a model in which mitochondrial and cytosolic Src act as non-canonical mediators of cardioprotection, distinct from RISK or SAFE pathways, but which can interact, at least with RISK/NO pathways [55]. While its localization at the inner mitochondrial membrane and the interaction with complex I are well documented [61, 65], the involvement of Src in GPCR-linked protective signaling, such as the apelin-APJ axis, underscores its multifaceted role. Further work is needed to clarify the relative contribution of mitochondrial *vs*. cytosolic Src pools and to determine whether these mechanisms extend to late IPC [43].

#### p38 MAPK as an additional pathway

Although not considered part of the canonical prosurvival cascades [75, 77, 81], p38 MAPK has been consistently implicated in both IPC and RIC. Its activation contributes to cardioprotection in rodents [157], rabbits [164], and pigs [196], while pharmacological blockade prevents RIC-mediated benefits in rats [70]. Importantly, isoforms appear to diverge in function: p38α activation during preconditioning triggers cardioprotection [200], yet its activation during sustained ischemia exacerbates injury [113]; in contrast, p38β activity has been associated with infarct size reduction in IPC [195, 196]. The dual role of p38MAPK is also evident in myocardial IRI. In this context, p38MAPK plays a temporally dynamic role: its early activation promotes inflammatory cytokine production, whereas later downregulation may protect against apoptosis and metabolic stress. The mechanisms driving this reduction remain unclear. Since p38MAPK influences GLUT4 expression and translocation, its decreased activity may impair glucose uptake in cardiomyocytes. Emerging evidence suggests that altered p38MAPK modulates MEF2 and AS160-Rab8a signaling and disrupts GLUT4-mediated metabolism, thus exacerbating myocardial injury during early reperfusion [152, 194]. Moreover, mitochondrial involvement is supported by studies showing p38 localization within cardiac mitochondria [14, 108]. In neonatal rat cardiomyocytes, mitochondrial p38β interacts with Mn Superoxide Dismutase, phosphorylating it at key residues, thereby reducing ROS and promoting survival, including in settings of 17β-estradiol–mediated protection [130, 138]. Both isoforms, however, display compartmentalized activity: during ischemia, mitochondrial p38 is activated via PKCε, whereas reperfusion induces activity in multiple cellular fractions [12, 14]. Non-selective pharmacological inhibition mitigates mitochondrial swelling, ROS formation, and loss of membrane potential when applied before ischemia, but fails if administered at reperfusion [114].

Despite strong experimental evidence, the dual and sometimes opposing effects of p38α and p38β, together with the lack of isoform-specific tools, complicate interpretation. Clinical translation remains uncertain, as illustrated by the neutral outcome of a trial with an oral p38 inhibitor in non-ST-elevation myocardial infarction [167].

*In summary*, mitochondrial p38 MAPK may act as a non-canonical protective pathway by modulating oxidative stress and mitochondrial integrity. Yet, its precise submitochondrial localization and isoform-specific roles remain unresolved, underscoring the need for further mechanistic studies [167].

#### PINK1-mediated mitophagy as a non-canonical cardioprotective pathway

Beyond the classical prosurvival cascades, PTEN-induced putative kinase 1 (PINK1) has emerged as a regulator of mitochondrial quality control through its role in mitophagy. Under basal conditions, PINK1 is rapidly imported and degraded within mitochondria, resulting in low steady-state levels [215]. When mitochondrial membrane potential collapses, this degradation is prevented: PINK1 accumulates on the outer mitochondrial membrane, undergoes autophosphorylation, and recruits the E3 ligase Parkin, which ubiquitinates outer membrane proteins, such as Mfn1, Mfn2, and VDAC, thereby promoting mitochondrial fission and selective clearance of damaged organelles [147, 202, 207, 257]. PINK1-independent mitophagy pathways have also been described [11, 57, 215].

Experimental data support a broader role for PINK1 in cardiomyocyte survival beyond mitophagy. Cardiomyocytes from PINK1-deficient mice exhibit reduced respiration, impaired membrane potential, and increased ROS production during simulated IR, leading to greater susceptibility to mPTP opening and cell death [201]. Accordingly, infarct size is exacerbated in PINK1 knockout hearts, whereas overexpression confers protection [201]. Similar findings are reported for Parkin-deficient mice subjected to left anterior descending coronary artery (LAD) ligation, which display higher mortality, reduced mitophagy, and impaired cardiac function [111]. Both in vivo and in vitro models of myocardial ischemia/reperfusion consistently show induction of PINK1 and Parkin [29, 90, 91], a process modulated by proteins, such as RhoA [213, 214] and microRNA-421 [223]. Nevertheless, contrasting reports describe reduced PINK1 levels in certain ischemia/reperfusion settings [31, 203], suggesting context-dependent regulation.

Although activation of autophagy is generally protective in IRI [193], excessive mitophagy may aggravate damage depending on the severity and duration of ischemia [209] (see also below). Evidence for PINK1 involvement in conditioning paradigms is indirect: while its activation has been observed in renal IPC [117] and cerebral RIC [94], studies in the heart remain limited. Pharmacological interventions, such as remifentanil [31], triiodothyronine [17], and acetylcholine [203] have been shown to enhance PINK1 expression and mitophagy, whereas aldehyde dehydrogenase 2 activation is associated with reduced PINK1 [90].

Taken together, PINK1 acts as a mitochondria-centered pathway of cardioprotection by modulating mitophagy, mitochondrial dynamics, and oxidative stress responses. However, direct evidence linking PINK1 activation to IPC, RIC, or PostC in the myocardium is lacking, highlighting the need for further studies to establish its role as a therapeutic target.

### Hippo-YAP signaling pathway as a novel modulator of cardioprotective pathways

The Hippo-YAP signaling pathway plays a vital role in controlling cell death, proliferation, and differentiation, which are central to cardiovascular disease progression and myocardial repair and regeneration [235]. YAP (Yes-associated protein), a key effector of this pathway, contributes to cardioprotection by interacting with molecular mechanisms that overlap with the established RISK and SAFE pathways.

The RISK pathway, mainly involving PI3K-Akt and ERK1/2 signaling, promotes cell survival during IRI by preventing apoptosis and mitochondrial dysfunction. YAP engages with various growth factors and transcription factors that activate downstream pro-survival kinases, complementing the protective effects of the RISK pathway. Notably, adult hearts have a limited capacity for cardiomyocyte renewal, which contributes to heart failure after myocardial infarction. In Hippo-deficient mouse models, injury induces Pitx2 expression, which is essential for cardiac repair. Pitx2 regulates genes related to mitochondrial function and ROS detoxification, acting in concert with YAP. Nrf2, a key antioxidant response regulator, controls Pitx2 expression and localization. Loss of Pitx2 results in increased ROS, but antioxidant treatment can rescue this effect, highlighting the pathway’s critical role in heart regeneration [208]. Genetically, YAP’s cooperation with Pitx2 reduces oxidative stress by lowering ROS levels. This is a major factor in reperfusion injury targeted by both RISK and SAFE pathways [208].

Concurrently, the SAFE pathway, characterized by JAK-STAT3 activation and anti-inflammatory signaling, converges with YAP-mediated processes. For example, YAP’s interaction with interleukin-37 (IL-37) inhibits the NLRP3 inflammasome in macrophages, reducing inflammation and mimicking the anti-inflammatory effects of cytokines, such as IL-10, typical of the SAFE pathway [25, 228]. Additionally, YAP/TEAD4-dependent induction of heat shock protein A12B (HSPA12B) promotes angiogenesis after myocardial infarction, supporting tissue repair and regeneration, a process also enhanced by RISK and SAFE signaling [48, 182].

Research on the Hippo signaling cascade has revealed only limited but growing insights into its influence over mitochondrial activity and structural remodeling [18, 127]. Central downstream elements, particularly YAP and TAZ, have been shown to fine-tune key bioenergetic processes, such as glutamine metabolism, the tricarboxylic acid cycle (TCA) cycle, and electron transport, with YAP–TEAD complexes directly enhancing genes that channel glutamine into α-ketoglutarate for fueling respiration. Through interactions with PGC-1α, the same axis also shapes mitochondrial biogenesis. Hippo activity critically dictates mitophagy: MST1, for example, restrains the protective PINK–Parkin program, while YAP can alter mitochondrial fission–fusion dynamics in tumor-like contexts. The MST1/β-Catenin/Drp1 branch favors fission, fragmentation, and cell death, whereas Hippo suppression and YAP upregulation bias mitochondria toward fusion. The pathway is further tied to oxidative stress handling: when Hippo is activated, ROS detoxification becomes impaired, and persistent ROS can in turn activate Hippo components, leading to apoptosis through Bax-mediated mitochondrial outer membrane permeabilization. Importantly, mitochondria are not only targets but also regulators, as ROS or stress signals can shift YAP/TAZ localization and activity via mechanisms involving RhoA oxidation, MOB1 acetylation, or mitochondrial proteins, such as EFHD1 and MIGA-2 [18]. These observations emphasize a reciprocal relationship between mitochondrial status and Hippo activity.

Together, these data underscore Hippo-YAP signaling as a crucial modulator of delayed cardioprotective pathways, coordinating cellular survival and anti-inflammatory responses following myocardial injury. Because this signaling crosstalk intersects with redox status and numerous other regulatory networks, dissecting its contribution under pathological conditions may open new directions for therapeutic innovation.

### NLRP3 inflammasome and pyroptosis: new targets in cardioprotection

Recent evidence points to the NLR family pyrin domain containing 3 (NLRP3) inflammasome as a key player in amplifying myocardial injury through sterile inflammation. NLRP3 activation promotes the maturation of pro-inflammatory cytokines, such as IL-1β and IL-18 and contributes to *pyroptotic cell death*—a relatively rapid inflammatory form of cell death. Mitochondria act as both targets and amplifiers of the NLRP3 inflammasome response: mitochondrial dysfunction leads to increased ROS production, mitochondrial DNA (mtDNA) release, and further NLRP3 activation, creating a vicious cycle [177, 211].

**Fig. 1:**
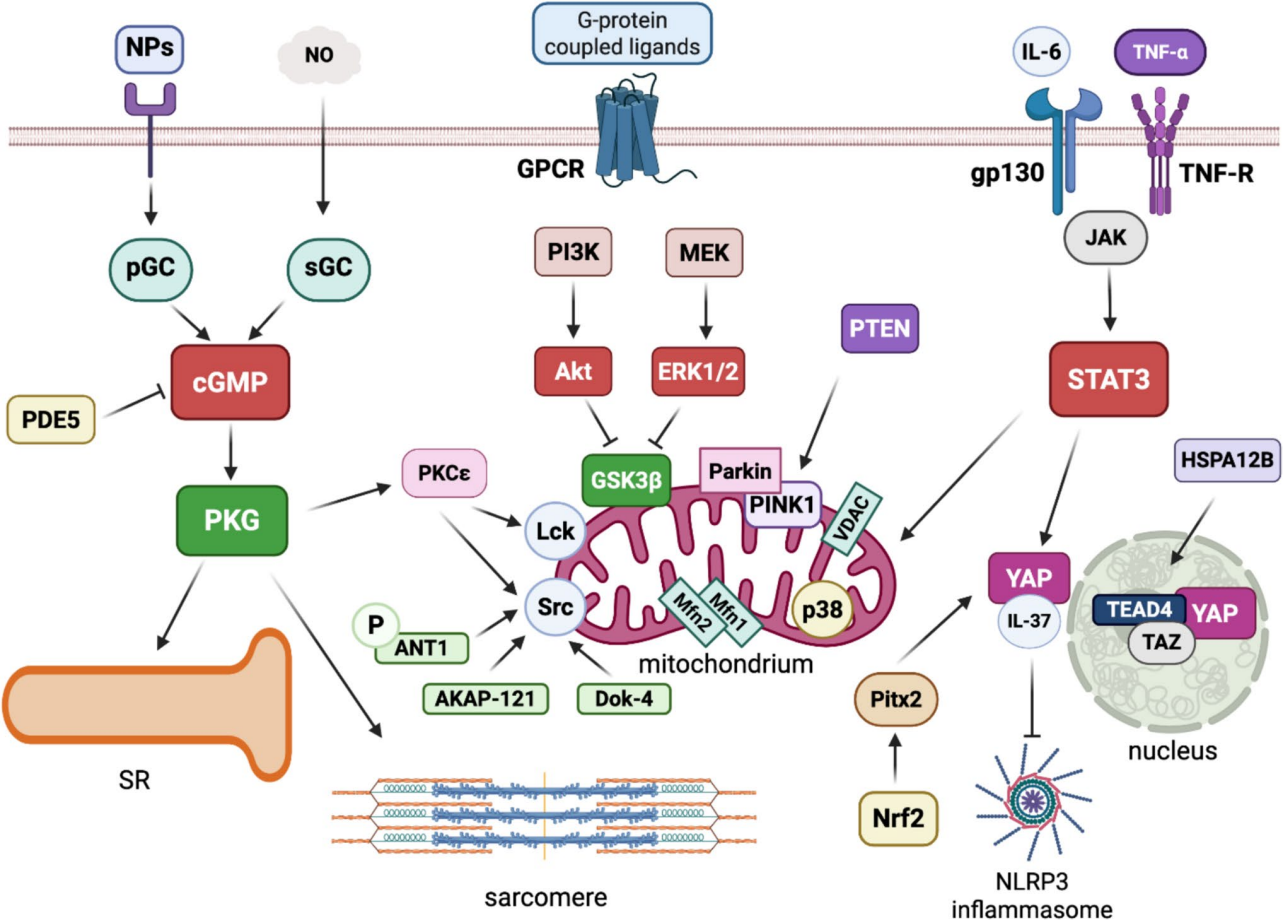
Schematic overview of canonical and non-canonical signaling pathways in cardioprotection. Canonical pathways include the RISK (Reperfusion Injury Salvage Kinase) pathway, the SAFE (Survivor Activating Factor Enhancement) pathway, and the NO/cGMP signaling axis, which converge on mitochondria to promote cell survival and limit IRI. In addition, several non-canonical pathways have emerged as complementary modulators of cardioprotective responses, such as the Src-family kinases (SFKs), p38 MAPK signaling, PINK1 and Hippo–YAP pathways, highlighting the complexity and interconnectivity of the molecular networks underlying myocardial protection. *AKAP-121* A-kinase anchoring protein 121, *Akt/PKB* protein kinase B, *ANT1* adenine nucleotide translocator 1, *cGMP* cyclic guanosine monophosphate, *Dok-4* docking protein 4, *ERK1/2* extracellular signal-regulated kinase 1/2, *gp130* glycoprotein 130, *GPCR* G-protein coupled receptor, *GSK-3β* glycogen synthase kinase-3beta, *HSPA12B* heat shock protein A12B, *IL-6* interleukin-6, *IL-37* interleukin-37, *JAK* janus kinase, *Lck* lymphocyte-specific protein tyrosine kinase, *MEK* mitogen-activated protein kinase kinase, *Mfn1* Mitofusin-1, *Mfn2* Mitofusin-2, *NLRP3* NLR family pyrin domain containing 3 protein, *NO* nitric oxide, *NPs* natriuretic peptides, *Nrf2* Nuclear factor erythroid 2–related factor 2, *p38* p38 mitogen-activated protein kinase, *PDE5* phosphodiesterase type 5, *pGC* particulate guanylate cyclase, *PI3K* phosphatidylinositol-3-kinase, *PINK1* PTEN-induced putative kinase 1, *Pitx2* pituitary homeobox 2, *PKC-ε* protein kinase C epsilon, *PKG* protein kinase G, *PTEN* phosphatase and tensin homolog, *sGC* soluble guanylyl cyclase, *SR* sarcoplasmic reticulum, Src proto-oncogene tyrosine-protein kinase, *STAT3* signal transducer and activator of transcription 3, *TAZ* tafazzin, *TEAD4* TEA domain transcription factor 4, *TNF-α* tumor necrosis factor α, *TNF-R* tumor necrosis factor receptor, *VDAC* voltage-dependent anion channel, *YAP* Yes-associated protein

Targeting the NLRP3 inflammasome has emerged as a promising strategy to limit IRI and enhance cardioprotection. Pharmacological inhibition or genetic deletion of NLRP3 reduces infarct size, attenuates inflammatory cytokine release, and preserves cardiac function in preclinical models. Moreover, NLRP3 blockade mitigates pyroptosis, thereby preventing excessive loss of viable cardiomyocytes. Recent studies also highlight the interplay between NLRP3 and key cardioprotective signaling pathways, including those mediated by cGMP and nitric oxide, suggesting that combined modulation of these pathways could synergistically improve outcomes [177]. Thus, therapies targeting the NLRP3 inflammasome offer a novel approach to dampen sterile inflammation and promote myocardial repair following ischemic injury (Fig. 1). For a deeper examination of the role of the NLRP3 inflammasome in cardioprotection, readers may consult several recent comprehensive reviews [177, 210, 211, 260].

## Different modalities of cell death: distinct targets?

Many of these molecular pathways described above are central to the processes leading to cell death. It merits emphasis that there are multiple forms of cell death and there is evidence that IRI triggers several of these. While necrosis and apoptosis are the classically described forms of cell death in myocardial IRI [9, 41, 77, 190], there are less commonly described regulated forms, including autophagy, autosis, ferroptosis, necroptosis, and parthanatos that cumulatively may contribute significantly to cell death and myocardial injury and represent promising therapeutic targets [79, 154]. Indeed, pharmacological or genetic inhibition of these mechanisms has demonstrated cardioprotective effects in preclinical models. The current challenge is to understand the temporal and spatial interactions among these cell death pathways and to identify context-specific targets that can be safely and effectively modulated in patients. In Table 2 and below, we briefly describe these various forms of cell death, and then we will consider the suggested targeting for each of them.

**Table 2:** Evidence of causal involvement of cell death pathways in cardioprotection

Cell death	Cell population(s) mainly involved/studied	Causal evidence (approaches)	Experimental model(s)	Experimental timing	Refs.
Autophagy/autosis	Cardiomyocytes	Glucose deprivation	Primary neonatal rat ventricular myocytes	24 h starvation	[148]
	Cardiomyocytes	NHE1 KO	Mouse I/R model	I: 30 min/R: 3 h or 24 h	[93]
Ferroptosis	Cardiomyocytes	LPS-induced cardiac injury	H9c2 cells	6 h treatment	[120]
Necroptosis	Cardiomyocytes	4-HNE	H9c2 cells; mouse I/R model	I: 30 min/R: 4 h or 24 h	[249]
	Cardiomyocytes	Necrostatin-1 and Z-VAD	Mouse I/R model	I: 30 min/R: 2 h or 4 h	[109]
	Cardiomyocytes	RIP1 inhibitor	Rat I/R model	I: 30 min/R: 40 min	[206]
	Cardiomyocytes	Cold ischemia	Rat cardiac allograft transplantation	4 h cold ischemia	[216]
Parthanatos	Endothelial cells	NAC and Nicotine	HAECs	24 h treatment	[238]
Pyroptosis	THP-1 cells	LPS and NLRP3 inhibitor	Human monocytic THP-1 cells	4 h treatment	[59]
	THP-1 cells	LPS and NLRP3 inhibitor	Human monocytic THP-1 cells	4 h treatment	[60]
	Cardiomyocytes	NLRP3 inhibitor	I/R rat model	I: 30 min/R: 20 or 60 min	[145]

*4-HNE* 4-hydroxy-2-nonenal, *H9c2* rat cardiomyoblast cells, *HAECs* human aortic endothelial cells, *I* ischemia, *I/R* ischemia/reperfusion, *LPS* lipopolysaccharide, *NAC* *N*-acetyl-l-cysteine, *NHE1* Na^+^/H^+^ exchanger, *NLRP3* NLR family pyrin domain containing 3 protein, *R* reperfusion, *RIP1* receptor-interacting protein 1, *THP-1* T-cell human promonocytic leukemia-1 cells

### Autophagy

Autophagy is not inherently a form of cell death. Nevertheless, it can be associated with cell death in certain contexts. It has different features in ischemia and reperfusion and may be detrimental or protective [3, 98, 118, 141–143]. During the ischemic phase, autophagy is rapidly activated as a compensatory mechanism in response to the imbalance in intracellular energy metabolism, depletion of ATP, and accumulation of damaged proteins and organelles. This process facilitates the degradation and recycling of cellular components, helping to maintain a minimal energy supply and limit further injury [118, 141, 142].

However, upon reperfusion, the role of autophagy becomes more complex and context-dependent. While moderate autophagy continues to exert protective effects—by removing dysfunctional mitochondria (mitophagy), reducing oxidative stress, and preventing apoptosis—its excessive activation can become detrimental. Uncontrolled or prolonged autophagy may lead to excessive degradation of essential cellular structures, thereby accelerating cell death and exacerbating myocardial injury [3, 98, 143]. This dual role underscores the importance of tightly regulated autophagic activity during ischemia and/or reperfusion to optimize cardioprotection while avoiding maladaptive effects.

#### Autosis

The development of this cell death modality is associated with dysregulated autophagy. It is individuated by electron microscopy and immunofluorescence tests and is characterized by mitochondrial alterations with nuclear depressions and fragmented endoplasmic reticulum [243]. In cardiac ischemia/reperfusion models, autosis has been observed to be caused by excessive accumulation of autophagosomes, thus suggesting that late-phase inhibition of autophagy after reperfusion could prevent autosis [148]. Recent studies have shown that autosis in ischemia/reperfusion models is reduced by cardiac glycosides, which, by reducing the action of Na^+^-K^+^-ATPase, reduce its interaction with Beclin1; the mechanism of interaction remains unknown at the moment [134]. An inhibitor of autosis is empagliflozin (EMPA), which mediates its inhibitory action by directly blocking the activity of NHE1. NHE1 is a recognized target for the protective action of EMPA; in fact, reduces the excessive formation of autophagosomes [93]. Consequently, EMPA acting on NHE1 reduces autosis and exerts a cardioprotective action. The protective action of EMPA was found in cardiomyocyte-specific NHE1 knockout mice (NHE1 cKO), and the target in this case was Beclin-1; in the same model, a reduction of cardioprotective autosis was obtained by inhibiting autophagy levels by targeting Beclin 1 but not NHE1. Furthermore, EMPA reduced autosis and exerted cardioprotective effects by reversing autophagic cell death caused by Tat-beclin1 or glucose deprivation in cardiomyocytes [93, 227].

### Ferroptosis

Ferroptosis is a non-apoptotic, iron-dependent cell death modality characterized by intracellular iron accumulation and lipid peroxidation, leading to rupture of cell membranes and subsequent cell death [44]. The causes of ferroptosis include altered iron metabolism, glutathione (GSH) deficiency, and reduced GPX4 activity, and lipid peroxidation. Ferroptosis is characterized by an increase in the production of ROS, which in turn causes lipid peroxidation, a central process capable of establishing a destructive cycle where interactions between lipid peroxidation products and iron ions increase oxidative damage to cellular membranes. Cellular antioxidant resources, such as GSH, are exhausted, accelerating the progression of ferroptosis [135]. A lower activity of GPX4, a GSH-dependent antioxidant enzyme, allows lipid peroxidation to proceed unhindered, consequently triggering ferroptosis [120]. Recent studies have shown that ferroptosis is activated by molecules, such as RSL-3 and elastin, which inhibit both GPX4 and the cystine/glutamate transporter [45]. Among the inhibitors studied to block ferroptosis, Ferrostatin-1 (fer-1) has recently been studied, whose mechanism of action is aimed at removing the alkoxy radical produced by the reduction of ferrous iron from lipid hydroperoxides. A second protective action of Fer-1 is its ability to produce a complex with Fe^+^, reducing the level of unstable cellular iron [153].

### Necroptosis

It is a form of regulated (programmed) cell death that morphologically resembles necrosis but is controlled, such as apoptosis. It is typically triggered when death receptors (such as TNF receptor 1) are activated, but the apoptotic pathway is blocked. It is highly proinflammatory because the loss of cell membrane integrity leads to the release of intracellular contents, which in turn activate the immune system and promote inflammation. Necroptosis is a tightly regulated process mediated by specific proteins, primarily RIPK1 (Receptor-Interacting Protein Kinase 1), RIPK3 (Receptor-Interacting Protein Kinase 3), and MLKL (Mixed Lineage Kinase Domain-, such as protein). These molecules work together to initiate and execute the necroptotic pathway when apoptosis is inhibited or unavailable. Among the activators of RIP1, the products of lipid peroxidation characteristic of reperfusion are indicated, in particular 4-hydroxy-2-nonenal (4-HNE) [249]. Once activated, this pathway leads to the rupture of the cell membrane, causing the release of intracellular contents into the surrounding tissue. This event does not go unnoticed by the body: it triggers an immune response, promoting inflammation. Due to these characteristics, necroptosis plays a significant role in the development and progression of several pathological conditions, such as IRI, neurodegenerative disorders, and various inflammatory diseases. It is often regarded as a harmful process in the context of disease, and inhibiting the necroptotic pathway is being actively explored as a potential therapeutic strategy for conditions associated with excessive inflammation [159]. In the heart, necroptosis has been implicated in various forms of IRI and depends on the activation of the RIPK1/RIPK3/MLKL pathway [2]. Inhibition of both necroptosis and apoptosis has been shown to enhance cardioprotection [109]. CaMKII (Ca^2+^/calmodulin-dependent protein kinase II), known to trigger inflammatory responses via NF-κB activation during IR, also contributes to necroptotic signaling. Inhibition of CaMKII has been found to suppress necroptosis and improve cardiac [128, 206]. Additionally, simvastatin pretreatment reduces RIPK1 and RIPK3 activity in rat cardiac allografts, further supporting necroptosis involvement [216]. MicroRNAs also modulate this process. miR-223, in particular, appears to protect the heart against IRI. In transgenic mice overexpressing miR-223, reduced activation of RIPK1/RIPK3/MLKL and the NLRP3 inflammasome was observed, whereas knockout mice showed increased susceptibility to necroptosis and inflammation after ischemia/reperfusion [47, 187].

### Parthanatos

It is characterized by the intervention of poly (ADP-ribosome) polymerase 1 (PARP-1) [50]. PARP-1 is an ADP-ribosyltransferase capable of transferring ADP-ribose from NAD^+^ to receptor proteins and was originally identified as a DNA nick sensor enzyme activated by DNA breaks [101]. PARP-1 activation, dependent on the degree of DNA damage, is induced by stresses capable of determining DNA fragmentation, such as ROS [189, 253]. When DNA damage is severe, hyperactivation of PARP-1 can be observed with consequent accumulation of poly (ADP-ribose) (PAR), and consumption of high amounts of NAD+. PAR can translocate under these conditions to the mitochondria causing the release of apoptosis-inducing factor (AIF) [36, 226]. The release of AIF from the mitochondria leads to the formation of a complex with macrophage migration inhibitory factor (MIF) at the cytoplasmic level. This AIF/MIF complex reaches the nuclear level, where it causes further DNA damage, such as condensation and fragmentation leading to cell death [92, 131, 225]. Recent studies have demonstrated the appearance of parthanatos in endothelial cells induced by various stimuli, such as ROS, ox-LDL, hyperglycemia and Ang II [122, 253]. At the endothelial level, the action of ROS can induce, as in other cellular models, death through various mechanisms, such as parthanatos and ferroptosis, as data from pathological animal models demonstrate. The action of an antioxidant, N-acetylcysteine (NAC), is able to block not only parthanatos but also other forms of cell death, such as ferroptosis, demonstrating the close correlation between the onset of these mechanisms and the production of ROS, key elements of IRI [238, 253].

### Pyroptosis

As described above, pyroptosis is induced by inflammation, specifically by NLRP3 inflammasome activation. Pyroptosis begins during the early stages of reperfusion and is responsible for acute IRI. It is induced by two pathways: the first, caspase-1 dependent or classical, and the second, non-classical, caspase-4/5/11 dependent mechanisms. Pyroptosis is characterized by DNA damage, cellular swelling, and consequent leakage of cellular contents, particularly pro-inflammatory cytokines, such as IL-1β and IL-18 [41, 46]. It has been described in cardiomyocytes and linked to cardiovascular diseases, such as myocardial infarction, heart failure, and atherosclerosis [169, 175, 210].

## The overlooked players in cardioprotection: vessels, blood, and the immune system

Importantly, recent advances suggest that cardioprotection should not be viewed as a phenomenon confined solely to cardiomyocytes, but rather as a complex, integrated process involving the coronary circulation, the vascular compartment, and the immune system. The coronary circulation itself represents both a primary target of IRI and a key determinant of tissue recovery, yet it has been relatively neglected in many studies [76, 79, 82, 86]. Expanding the analysis to include microvascular dysfunction, endothelial injury, and the no-reflow phenomenon is therefore essential to a more comprehensive understanding of myocardial protection. Similarly, in the context of RIC, limited progress may reflect not only oversimplified models of cardiac signaling but also our incomplete understanding of signal transduction from the periphery to the heart [107]. Increasing evidence points to a complex neurohumoral network involving multiple organs, with the spleen emerging as a central relay in the propagation of cardioprotective signals [84, 124]. Within the circulation, protective signals appear to be conveyed through both plasma components and blood cells, a process that may be modulated or disrupted by pharmacological agents [105, 125]. Furthermore, the delayed (second) window of protection, traditionally attributed to gene expression and protein synthesis, may also involve vascular mechanisms and interactions between the heart and immune system, including crosstalk with remote organs, such as the spleen. These emerging concepts underscore the need to broaden the mechanistic framework of cardioprotection to include the vasculature and the immune system, encompassing both innate and adaptive responses [8, 33].

## Emerging targets for cardioprotection

All the above-described overlooked players and cell death mechanisms represent targets for cardioprotection. In a recent review, we have described a couple of promising mechanical approaches, such as ventricular unloading and SSO_2_ (SuperSaturated Oxygen), which have some application in the clinic, but deserve more attention [230]. In that review, we have also described some consolidated pharmacological approaches, such as beta-blockers [230]. Here, we describe several emerging pharmacological and non-pharmacological approaches targeting some of the overlooked players and cell death mechanisms.

### Macrophages and inflammation

Inflammation and macrophages, central effectors of innate immunity, have emerged as pivotal modulators in cardiovascular disease, and they are also interesting targets in cardioprotection.

The functions of macrophages are largely determined by their different activation states. Typically, they are divided into two main types: M1 macrophages, which are classically activated and involved in *pro-inflammatory* responses, and M2 macrophages, which are alternatively activated and help with tissue repair and *anti-inflammatory* processes. However, recent research has shown that macrophages can adopt additional phenotypes depending on the signals they receive. The variety of receptors and molecules on their surface—including pattern recognition receptors (PRRs), cytokine receptors, and chemokine receptors—plays a key role in controlling their differentiation, activation, and ability to engulf pathogens or debris (phagocytosis). This diversity allows macrophages to respond flexibly to different signals in their environment. The contribution of macrophages to cardiac homeostasis and pathology is highly context-dependent: protective when M1/M2 polarization is balanced, yet potentially detrimental when skewed toward M1 pro-inflammatory phenotypes. Through surface receptors, macrophages sense danger signals from stressed or dying cardiomyocytes, orchestrating immune responses that are essential for maintaining intracellular and tissue homeostasis.

In the heart, macrophages regulate inflammation and lipid oxidation, contributing to both contractile dysfunction and electrical remodeling. During IRI, macrophages exert a dual function: M1 macrophages clear cellular debris, whereas M2 macrophages facilitate tissue repair and remodeling [236]. Moreover, macrophages can modulate cardiac electrophysiology directly by secreting neurotrophic factors, such as nerve growth factor (NGF) and amphiregulin (AREG) and forming connexin-mediated electrical connections with cardiomyocytes, thereby reducing arrhythmic risk. Pathophysiological stimuli, such as angiotensin II or deoxycorticosterone acetate (DOCA), activate macrophages in hypertension, promoting vascular remodeling and fibrosis through crosstalk with cardiac fibroblasts and regulation of extracellular matrix turnover, cytokines (IL-1β, IL-6, TNF-α), and metalloproteinases [185]. Given this central role, macrophages are increasingly recognized as attractive targets for immunomodulatory therapies. Among promising strategies, C–C chemokine receptor type 2 (CCR2) antagonism reduces monocyte recruitment to inflamed myocardium, dampening M1-driven inflammation, while Toll-like receptor 4 (TLR4) inhibition can prevent pro-inflammatory cytokine release and foam cell formation in atherosclerosis [231]. Additionally, promoting the M2 phenotype through IL-38 has been shown to mitigate inflammation and improve post-infarction cardiac function [121].

Emerging cardioprotective strategies increasingly exploit the interplay between regulated cell death and inflammation. Inhibitors of the NLRP3 inflammasome not only block pyroptosis (see above) but also limit the release of pro-inflammatory cytokines, conferring robust protection in preclinical ischemia/reperfusion models [59, 210, 211]. Nevertheless, whether inhibitors of the NLRP3 inflammasome target directly cardiomyocytes or macrophages to limit pyroptosis is a matter of controversy [63]. In our opinion, a persistent cardioprotective strategy should target both cell types. Therefore, inhibitors capable of penetrating both cell types should be preferred.

Similarly, agents targeting necroptosis (necrostatins), ferroptosis (liproxstatin-1), and parthanatos (PARP-1 inhibitors) attenuate cardiomyocyte death while curbing secondary inflammatory responses. These findings underscore the dual benefit of therapies that simultaneously restrain cell death and modulate inflammation, highlighting underexplored avenues for cardioprotection.

*Translational perspective:* Potential upstream pharmacological targets within the macrophage–inflammation axis include chemokine receptors (e.g., CCR2), pattern recognition receptors, such as TLR4, and inflammasome components (i.e., NLRP3), whereas downstream interventions may focus on cytokine signaling, macrophage polarization, and regulated cell death pathways. Notably, CCR2 antagonists, TLR4 inhibitors, PARP inhibitors, and NLRP3-targeting compounds have reached clinical evaluation in inflammatory or cardiovascular settings, albeit with variable and often indirect evidence for cardioprotection [175, 177, 210, 211, 230]. In contrast, strategies aimed at selectively modulating macrophage phenotypes or targeting necroptosis and ferroptosis remain largely supported by preclinical data, underscoring the need for translational studies to define their clinical relevance in myocardial ischemia/reperfusion injury.

### Targeting cell death mechanisms

#### Targeting autophagy and autosis

Targeting autophagy for therapeutic purposes requires careful consideration of both timing and magnitude. Interventions during the ischemic phase may aim to enhance autophagy transiently to maximize cellular energy recycling and clearance of damaged organelles, whereas modulation during early reperfusion should focus on preventing excessive activation that could lead to cell death. Potential targets include key regulators, such as mTOR, AMP-activated protein kinase (AMPK), and Beclin-1, which coordinate autophagic flux [96, 158, 250]. Pharmacological agents, such as rapamycin [40], metformin [250], or resveratrol [133] have been shown to stimulate protective autophagy, while inhibitors, such as 3-methyladenine or chloroquine, could be employed to restrain maladaptive autophagy during reperfusion [58]. Optimizing the timing and dosage of these interventions may offer a promising strategy to enhance cardioprotection in IRI.

Targeting autosis in IRI requires interventions focused on late-phase autophagy, when excessive autophagosome accumulation drives this form of cell death. As seen above, key molecular targets include Beclin-1, which regulates autophagosome formation, and NHE1, whose inhibition prevents maladaptive autophagic cell death. Pharmacological strategies include: (a) EMPA [93, 227], which may inhibit NHE1, reduce autosis, and preserve cardiomyocyte survival; and (b) cardiac glycosides, which decrease the interaction between Na^+^/K^+^-ATPase and Beclin-1, thereby limiting autosis. Timing and dosing are critical: treatments should be applied after reperfusion to restrain maladaptive autophagy without interfering with earlier protective processes. This approach offers a potential cardioprotective strategy by specifically reducing autosis in myocardial IRI and warrants further investigation.

#### Targeting ferroptosis

Targeting ferroptosis in myocardial IRI focuses on limiting lipid peroxidation, controlling intracellular iron, and restoring antioxidant defenses. Key targets include GPX4, whose activity prevents uncontrolled lipid peroxidation, and cellular iron pools, whose dysregulation drives ROS-mediated membrane damage. Pharmacological inhibitors, such as Ferrostatin-1 (Fer-1), act by scavenging lipid-derived radicals and chelating labile Fe^2+^, thereby reducing oxidative stress and ferroptotic cell death [153]. Early intervention, ideally during reperfusion, is critical to prevent the amplification of lipid peroxidation and cellular injury. Additional strategies may include boosting GSH levels or modulating cystine/glutamate transporters to enhance cellular antioxidant capacity [135]. Optimizing timing and dosage of these interventions represents a promising approach to limit ferroptosis-mediated myocardial injury.

#### Targeting necroptosis

Targeting necroptosis in IRI focuses on inhibiting key mediators of the receptor-interacting serine/threonine-protein kinase (RIPK)1/RIPK3/ mixed lineage kinase domain-like pseudokinase (MLKL) pathway to prevent cell death and limit inflammation. Potential interventions include pharmacological inhibition of RIPK1 (e.g., necrostatins) or RIPK3, as well as modulation of MLKL activity to block execution of necroptosis. Additional targets include CaMKII, whose inhibition reduces NF-κB–mediated inflammatory signaling and necroptotic activation, and microRNAs, such as miR-223, which downregulate RIPK1/RIPK3/MLKL and NLRP3 inflammasome activity. Timing of intervention is critical: targeting these pathways during early reperfusion may prevent excessive membrane rupture, immune activation, and subsequent inflammatory damage. Preconditioning strategies, such as simvastatin pretreatment, have also been shown to reduce RIPK1/RIPK3 activity, representing a potential cardioprotective approach [216, 256]. Optimizing dosage and timing of these interventions could effectively limit necroptosis-mediated myocardial injury while preserving adaptive cell responses.

#### Targeting parthanatos

Targeting parthanatos in IRI involves inhibiting PARP-1 hyperactivation and controlling downstream effectors, such as AIF and MIF, to prevent NAD^+^ depletion, mitochondrial dysfunction, and DNA fragmentation. Pharmacological inhibitors of PARP-1, including veliparib and olaparib, have been shown to reduce parthanatos-mediated cell death in preclinical models [132]. Currently, several clinical trials are evaluating PARP-1 inhibitors for the treatment of acute ischemic stroke, ischemic acute kidney injury, myocardial ischemia, diabetes and related cardiovascular complications, shock, and traumatic central nervous system injuries [e.g., JPI-289, Phase 2, NCT03062397 [88, 103]. Antioxidants, such as NAC, can also mitigate ROS-induced DNA damage, thereby limiting PARP-1 overactivation and subsequent parthanatos, while simultaneously reducing other ROS-dependent cell death mechanisms, such as ferroptosis [238, 253]. Timing of intervention is critical, with early application during reperfusion being most effective to prevent irreversible DNA damage and mitochondrial injury. Optimizing the dose and window of administration may enhance cardioprotection while preserving physiological DNA repair processes.

#### Targeting pyroptosis

Targeting pyroptosis in IRI focuses on inhibiting the activation of key inflammatory caspases and blocking the release of proinflammatory cytokines. Potential targets include caspase-1 in the canonical pathway and caspase-4/5/11 in the non-canonical pathway. Pharmacological inhibitors, such as VX-765 (caspase-1 inhibitor) or specific caspase-4/5/11 blockers, can reduce cytokine release, cellular swelling, and tissue inflammation. Modulation of upstream inflammasome components, such as NLRP3, represents an additional strategy to prevent pyroptotic cell death [175, 230]. In this context, our research group has conducted studies on the role of the NLRP3 inflammasome in myocardial IRI and the therapeutic potential of inflammasome inhibitors. In particular, we have developed and tested preclinical compounds, such as INF-4E, INF195, and INF200, which reduce NLRP3 activation, improve post-ischemic cardiac function, and decrease infarct size [59, 60, 145]. Timing is critical: interventions during early reperfusion are most effective in limiting acute tissue injury and dampening the inflammatory cascade. Optimizing the dose and window of these inhibitors may provide a promising approach to mitigate pyroptosis-mediated damage in IRI.

*Translational perspective:* Targeting regulated cell death pathways in myocardial IRI offers multiple pharmacological entry points, ranging from upstream modulation of nutrient- and stress-sensing pathways (e.g., mTOR, AMPK, PARP-1, inflammasome components) to downstream inhibition of executioner mechanisms, such as lipid peroxidation, membrane rupture, and inflammatory caspase activation. Among these approaches, NLRP3-, SGLT-, and PARP-inhibitors represent the most clinically advanced strategy (see below paragraph 6.1), having reached clinical evaluation in ischemic and cardiovascular settings, while agents targeting autophagy/autosis, ferroptosis, necroptosis, and pyroptosis remain largely supported by preclinical evidence. Importantly, the efficacy of these interventions is highly dependent on the timing of delivery, with early reperfusion emerging as a critical therapeutic window. These considerations support the development of rational, multi-target strategies aimed at simultaneously limiting cardiomyocyte death and secondary inflammation, while future translational studies are needed to define safety, optimal dosing, and clinical relevance of cell death–targeted therapies in myocardial ischemia/reperfusion injury.

### Targeting mitochondria

Mitochondria play a central role in myocardial ischemia/reperfusion injury, acting not only as energy providers but also as key regulators of regulated cell death. Accordingly, mitochondria-targeted cardioprotection offers multiple pharmacological entry points, ranging from upstream modulation of mitochondrial dynamics and quality control—such as DRP1- and mitofusin-2–dependent fission–fusion processes—to downstream inhibition of mitochondrial permeability transition and bioenergetic collapse. Pharmacological inhibition of the mitochondrial permeability transition pore (mPTP), including cyclosporine A and novel cyclophilin D blockers, represents the most clinically advanced strategy within this field, although clinical outcomes have been variable and strongly context-dependent [28, 232]. In contrast, interventions targeting mitochondrial dynamics, mitochondrial signaling, or mitochondrial transfer remain largely supported by preclinical evidence. These observations highlight the importance of precise timing of intervention, particularly during early reperfusion, as well as improved patient stratification and rational combination strategies integrating mitochondrial protection with anti-inflammatory or cell death–targeted therapies to enhance translational success.

**Fig. 2:**
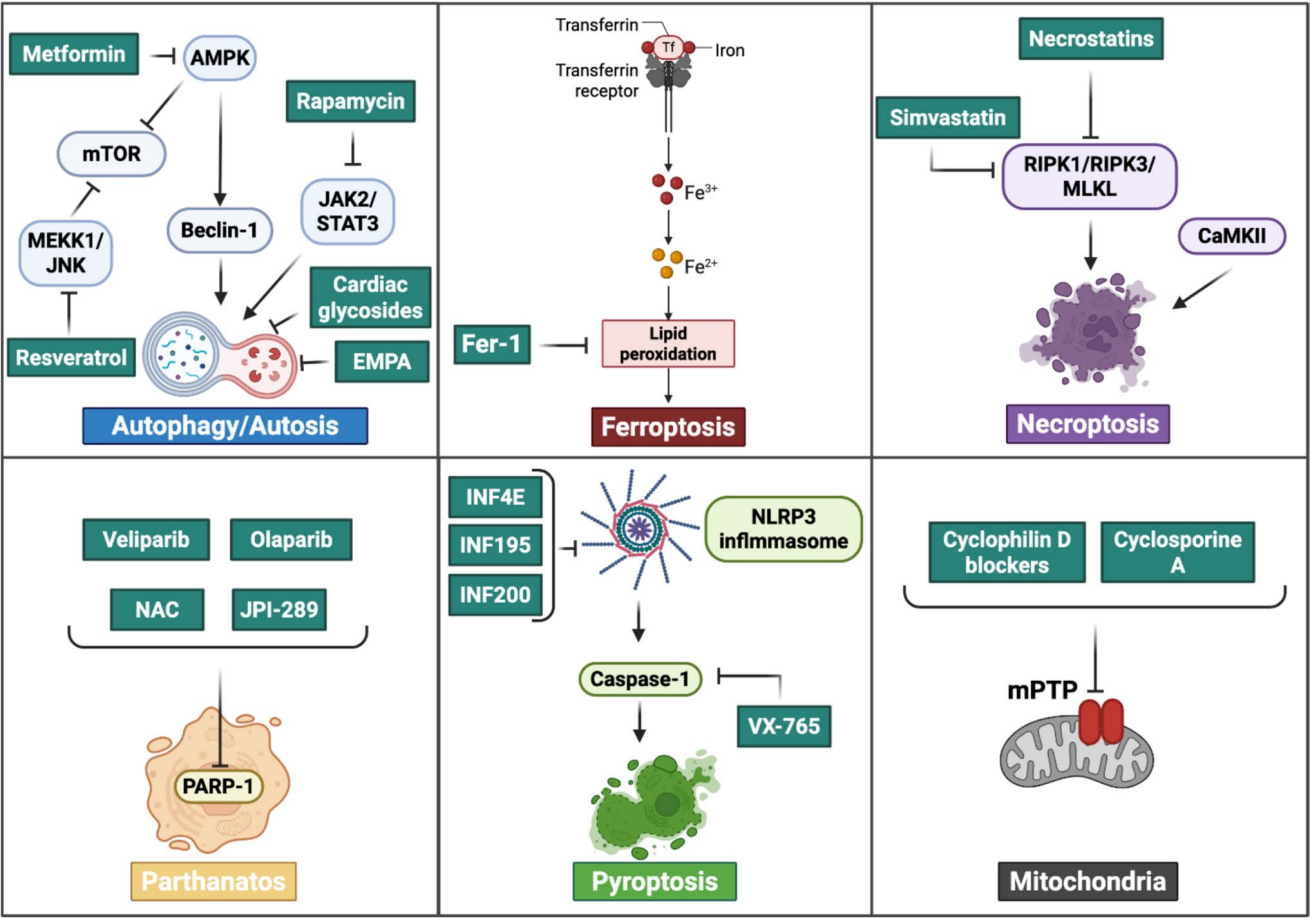
Schematic representation of compounds and drugs involved in cardioprotection, highlighting their molecular targets. The targets illustrated include autophagy and autosis, ferroptosis, necroptosis, parthanatos, pyroptosis, and mitochondria. Arrows indicate the modulation of each pathway by specific compounds, emphasizing their potential therapeutic roles in cardioprotection. *AMPK* AMP-activated protein kinase, *CaMKII* calcium/calmodulin-dependent protein kinase II, *JAK2* janus kinase 2, *JNK* jun N-terminal kinase, *MEKK1* mitogen-activated protein kinase kinase kinase 1, *MLKL* mixed lineage kinase domain-like protein, *mPTP* mitochondrial permeability transition pore, *mTOR* mammalian target of rapamycin, *NLRP3* NLR family pyrin domain containing 3 protein*, PARP-1* Poly(ADP-ribose) polymerase 1, *RIPK1* receptor-interacting protein kinase 1, *RIPK3* receptor-interacting protein kinase 3, *STAT3* signal transducer and activator of transcription 3

The identification of druggable targets within cardioprotective pathways has opened new avenues for therapeutic intervention. Above we have recapitulated some of the druggable strategies and discussed novel approaches, many of which still require validation in the clinical setting (Fig. 2). Below we analyze some additional approaches, including targeting of macrophages and inflammation as emerging targets for cardioprotection, and then we describe mitochondria transfer as a novel therapeutic approach in cardioprotection, which merits further study.

## Novel pharmacological agents and interventions in myocardial IRI

### Targeting innate and adaptive immunity: NLRP3-, SGLT- and PARP-inhibitors

**NLRP3 inhibitors** are emerging as a new class of immunomodulatory compounds with potential relevance across multiple inflammatory and cardiometabolic conditions, and their therapeutic potential continues to expand as increasing preclinical and translational evidence clarifies their mechanisms of action. For instance, an NLRP3 inhibitor, INF 195, protects endothelial cells from oxidative and lipotoxic stress by preserving viability, reducing pyroptosis, and maintaining angiogenic function [176]. Several recent reviews on NLRP3 inhibitors by us [175, 177] and others [210, 211] have been published, and some of the targets discussed therein are mentioned above.

Beyond direct NLRP3 inhibition, additional agents with anti-inflammatory and cardioprotective actions, namely SGLT2 antagonists and PARP inhibitors, have emerged as complementary therapeutic strategies in myocardial IRI.

**SGLT2 inhibitors** (e.g., EMPA, Dapagliflozin (DAPA), Canagliflozin) were developed as effective drugs in the management of patients with type 2 diabetes (T2D) [6]. Besides a number of preclinical studies [6, 168], several clinical studies and trials (e.g., EMPA-REG, CANVAS, and Declare-TIMI) have demonstrated their cardioprotective effect, highlighting a reduction in cardiovascular mortality and rehospitalization in patients with both T2D and heart failure [166, 234, 258]. Observations in diabetic and non-diabetic experimental models have demonstrated a reduction in myocardial ischemic damage, improved cardiac function, and a reduction in the development of heart failure while also preventing cardiomegaly, arrhythmia, and fibrosis. SGLT2 inhibition also improves endothelial function, myocardial energy metabolism, and reduces oxidative stress [6, 217]. SGLT2 inhibitors also possess anti-inflammatory properties and can reduce murine atherosclerosis [165]. In LPS-stimulated cardiac and immune cells, SGLT2 inhibition reduces the release of IL-1, IL-6, or tumor necrosis factor-α (TNF-α) and also preserves structural homeostasis with stabilization of the integrin α5-desmocollin-2 [186, 239].

The cardioprotective action exerted by SGLT2 inhibitors has several proposed mechanisms of action, including the reduction of intracellular Na^+^ and Ca^2+^, activation of STAT3 and AMPK, and inhibition of CamKII and NHE [7, 162, 168].

The role of PARP and the use of **PARP inhibitors** in parthanatos have been considered above. In brief, PARP involvement in myocardial IRI derives from the fact that PARP-1 is activated by DNA damage and oxidative stress, catalyzing the transfer of ADP-ribose units from NAD^+^ to target proteins. While transient activation supports DNA repair, PARP hyperactivation leads to excessive NAD^+^ and ATP consumption, resulting in metabolic collapse, mitochondrial dysfunction, and cell death by parthanatos, necrosis, or apoptosis under conditions of oxidative stress and ischemia/reperfusion (I/R) injury [139, 204]. During myocardial IRI, increased ROS production induces DNA strand breaks and sustained PARP overactivation in cardiomyocytes and endothelial cells, thereby aggravating energy depletion, inflammatory signaling, microvascular dysfunction, and infarct expansion. In this setting, PARP inhibitors preserve intracellular NAD^+^ and ATP levels, attenuate oxidative and inflammatory damage, and limit cardiomyocyte death, leading to reduced infarct size and improved post-ischemic functional recovery in experimental models. Pharmacological PARP inhibition (e.g., PJ34, 5-aminoisoquinoline) consistently confers cardioprotection by reducing necrosis and apoptosis, suppressing pro-inflammatory cascades, and improving endothelial function, partly through preservation of cellular bioenergetics and modulation of PI3K/Akt signaling [49, 54, 137, 205].

Beyond experimental IRI, PARP inhibitors have gained interest in cardiovascular medicine due to their ability to counteract oxidative stress-driven tissue damage, with beneficial effects reported across multiple cardiovascular pathologies [66, 102]. Although currently approved for oncological indications, their potential role in ischemic heart disease remains to be established and warrants further translational and clinical investigation.

### Mitochondria transplantation in cardioprotection

Beyond conventional pharmacological approaches, alternative strategies, such as mitochondrial transplantation and extracellular vesicles (EVs) derived from various cell types (e.g., mesenchymal stromal cells, platelets, endothelial cells, or cardiac progenitors), provide promising ways to deliver protective signals and modulate immune responses.

While the role of EVs has been extensively reviewed by us and others [4, 13, 38], here we focus specifically on mitochondrial transplantation as a novel approach to cardioprotection.

Mitochondrial dysfunction plays a central role in myocardial IRI, with functional alterations that begin during ischemia and worsen after reperfusion, leading to reduced myocardial contractility and cell survival [255]. Direct mitochondrial transplantation has emerged as a promising cardioprotective strategy, delivered via direct myocardial injection, intracoronary infusion, or intercellular transfer through mesenchymal stem cells exploiting connexin-43-rich gap junctions [39, 146, 149, 188]. Among these, intracoronary administration appears most suitable in clinical settings, especially during percutaneous coronary intervention, allowing mitochondria to be injected at reperfusion with significant protective benefits [19, 64, 149]. We acknowledge that this technique has several limitations and must be approached with caution [16]. Nevertheless, it warrants careful attention from researchers to fully establish its potential utility in clinical settings. The timing of delivery is crucial, as administration before ischemia mimics preconditioning, while delivery before or during reperfusion enhances ATP recovery, reduces infarct size, and improves ejection fraction in preclinical models [99, 146]. Beyond direct transplantation, nanotechnologies, such as the MITO-Porter liposomal system, offer a means of targeted mitochondrial delivery, fusing with mitochondrial membranes to restore function, and have shown promise in doxorubicin-induced cardiomyopathy models [1, 240].

Parallel to these approaches, non-coding RNAs (ncRNAs)—including miRNAs, long non-coding RNAs (lncRNA), and circular ribonucleic acid (circRNA)—are emerging as biomarkers and modulators of IRI, influencing mitochondrial dynamics, apoptosis, and autophagy [144]. Importantly, endoplasmic reticulum (ER)-mitochondria microdomains have been implicated as key regulators of oxidative stress and cell death pathways, with epigenetic control by ncRNAs shaping this crosstalk [219, 254]. Thus, therapeutic strategies that target both mitochondria directly and the ER/mitochondria interface may be necessary to achieve robust cardioprotection in IRI.

### Intracoronary hypothermia to reduce IRI

Another technique, recently proposed for limiting myocardial IRI is the selective intracoronary hypothermia. This technique, as described by Otterspoor et al. [171], starts by crossing the culprit lesion with a guidewire and advancing an over-the-wire balloon to the occlusion site, which is then inflated. A sensor-tipped wire is advanced distal to the balloon to monitor coronary pressure and temperature. During the occlusion phase, saline at room temperature is infused distal to the balloon (typically for ~ 7–10 min at 10–30 mL/min) to lower the distal coronary temperature by approximately 6–8 °C relative to core body temperature. After balloon deflation (reperfusion phase), the infusion is switched to cold saline (≈ 4 °C) and continued (commonly for ~ 10 min) to maintain the distal coronary temperature at roughly 4–6 °C below body temperature while reperfusion and warm coronary blood flow are present. Several preclinical and subsequent clinical feasibility studies have evaluated the safety and potential efficacy of this intracoronary cooling approach in acute coronary syndrome patients [184].

Current evidence from both systemic and intracoronary hypothermia studies provides little support for their routine use in STEMI patients undergoing primary PCI, as most trials have reported neutral outcomes regarding infarct size reduction and cardioprotection [184]. While animal studies initially suggested that cooling could mitigate IRI, these findings have not translated into consistent clinical benefits. One proposed explanation is that therapeutic hypothermia must be initiated before reperfusion to be effective, a strategy more feasible in experimental settings than in real-world clinical scenarios [140].

Past research efforts have been hampered by several methodological limitations, including non-standardized cooling protocols, delayed achievement of target temperatures, prolonged ischemia times, variable endpoints for myocardial injury, heterogeneous patient populations, and insufficient evaluation of cooling duration and rewarming strategies. Even though later trials have addressed some of these shortcomings, additional challenges remain, such as defining optimal patient selection, timing, and possible combination with pharmacological or invasive cardioprotective methods [174, 184]. Recent consensus recommendations on standardized endpoints for cardioprotection studies may guide future research toward more rigorous designs [23]. Ongoing trials, such as STEMI-Cool (NCT06128993), aim to clarify whether improved protocols and technologies can overcome previous barriers and eventually demonstrate a meaningful clinical benefit of hypothermia in the setting of acute coronary syndromes.

## Translational perspectives and future directions

### Challenges

Preclinical studies have identified many molecular targets and protective strategies for heart injury. Yet, turning these findings into effective treatments for patients has been difficult. One main reason is the difference between controlled lab models and the complex reality of human heart disease. Factors, such as comorbidities, co-medications, and variable reperfusion strategies, often prevent promising therapies from working in clinical trials [23, 83, 85].

Timing is critical: a key issue concerns the rapid onset of irreversible IRI, which occurs so quickly after reperfusion that, for example, intervention affecting transcription-dependent protein synthesis would likely be too slow to have an effect. Nonetheless, this does not reduce the significance of studies on these factors. For instance, transcription factors can exert other immediate actions: STAT3 has been shown to limit infarct size shortly after activation by directly influencing mitochondrial function in mice, rats, and pigs. Some interventions help only if given at the right moment. For example, boosting autophagy can be protective during ischemia but harmful if prolonged during reperfusion. Inhibitors of autosis or ferroptosis work best after reperfusion. Similarly, macrophages and inflammation can be both helpful and harmful, so careful modulation is needed. Another challenge is that cell death and inflammatory pathways are interconnected. Targeting a single pathway may not be enough. Multimodal approaches, which reduce cell death, control inflammation, and support mitochondria, are likely to be more effective, but need to be ascertained in clinical real life [32, 42].

### Emerging translational strategies

- ***Novel pharmacological approaches:*** These include SGLT2 inhibitors, PARP inhibitors, necroptosis blockers [216], ferroptosis inhibitors [153], and NLRP3-targeting compounds [59, 60, 145], which have demonstrated significant efficacy in experimental models and, in selected settings, preliminary benefits in clinical studies [153, 216]. Similar benefits have been obtained with SSO₂ therapy, RAS inhibitors, and calcium channel blockers [230].
- ***Cell- and organelle-based approaches*:** Mitochondrial transplantation [39, 146, 149, 188], extracellular vesicles [4, 13, 38], and non-coding RNAs [144, 219] can deliver protective signals, restore energy balance, and regulate inflammation. Basic studies and early-phase human trials are needed to test safety and efficacy.
- ***Mechanical and device-based approaches:*** These include *mechanical unloading* of the left ventricle to lower oxygen demand, edema, and oxidative stress during reperfusion [230]; *ischemic conditioning*, involving brief episodes of ischemia to activate protective signaling pathways; *gradual reperfusion* or postconditioning techniques, which modulate flow restoration to reduce microvascular injury [172, 180, 230, 252], and selective *intracoronary hypothermia* [140, 171, 184], which maintains the myocardial temperature below core temperature during reperfusion to limit IRI.

These strategies aim to mitigate the severity of IRI and potentially improve long-term cardiovascular outcomes. By targeting key mechanisms underlying IRI, they aim to attenuate cell death, inflammation, microvascular injury, and infarct size, with the ultimate goal of translating these effects into a reduction in downstream MACE in clinical settings. Careful optimization of dosing and timing, together with appropriate patient selection, will be critical to achieving meaningful clinical benefit.

### Future research directions

- ***Personalized cardioprotection:*** Stratifying patients according to prior treatments, genetic background, metabolic status, or inflammatory profiles may enable therapies to target the pathways most relevant to each individual. Biomarkers, such as circulating miRNAs, even within extracellular vesicles, or markers of inflammasome activation could guide treatment selection and monitor therapeutic efficacy [56, 156].
- ***Multi-target strategies and timing of delivery:*** Future studies should identify optimal therapeutic windows, particularly during early reperfusion, and determine whether combining cell death inhibitors with anti-inflammatory, mitochondrial-protective therapies, or device-based approaches can provide additive or synergistic benefits in real-world clinical settings [42, 178].
- ***Clinical translation:*** Well-designed preclinical studies and early-phase clinical trials should be conducted to assess safety and efficacy, in accordance with established guidelines [23].
- ***Advanced technologies:*** Nanocarriers, MITO-Porter systems, and gene therapy approaches may improve targeted delivery and reduce off-target effects [240].
- ***Systems biology and modeling:*** Systems biology approaches and computational modeling can integrate cell death, inflammation, and mitochondrial dynamics to predict therapeutic responses and optimize combination strategies [115].

## Conclusion

**Fig. 3:**
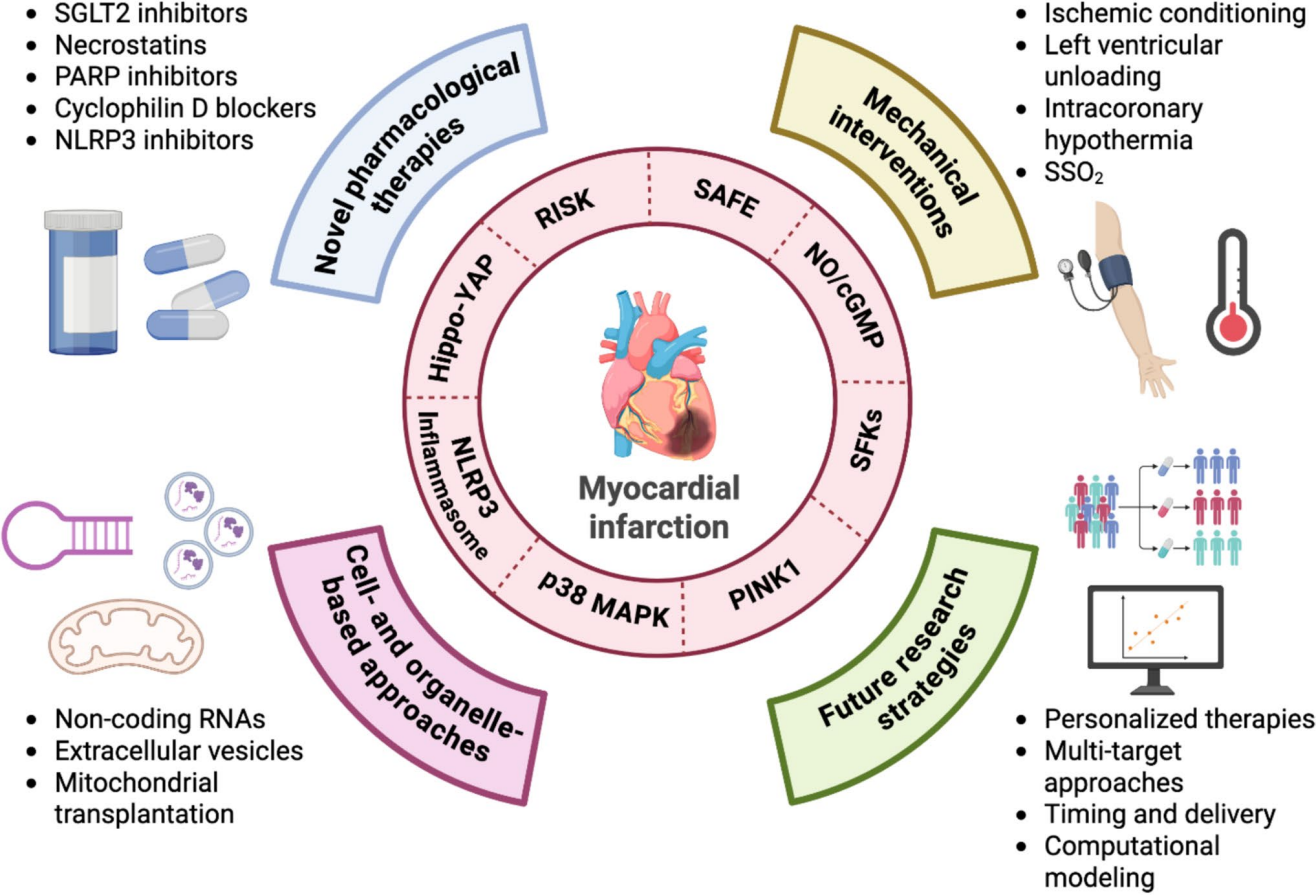
Graphical overview of emerging cardioprotective strategies in myocardial infarction (MI). The central panel illustrates key intracellular signaling pathways involved in IRI and cardioprotection, including RISK, SAFE, NO/cGMP, SFKs, PINK1, p38 MAPK, Hippo–YAP, and the NLRP3 inflammasome. Surrounding segments summarize major therapeutic domains under investigation. Novel pharmacological therapies include SGLT2 inhibitors, necrostatins, PARP inhibitors, cyclophilin D blockers, and NLRP3 inhibitors. Mechanical interventions comprise ischemic conditioning, left ventricular unloading, intracoronary hypothermia, and supersaturated oxygen (SSO₂). Cell- and organelle-based approaches involve non-coding RNAs, extracellular vesicles, and mitochondrial transplantation. Future research strategies emphasize personalized therapies, multi-target approaches, optimization of timing and delivery, and computational modeling

Moving cardioprotective strategies into clinical use calls for an integrated and multi-level approach. The chances of success rely on careful control of treatment timing, the ability to identify subgroups of patients most likely to benefit, and the adoption of advanced methods for drug and signal delivery [42, 115, 178, 240]. Despite major advances in both preclinical studies and clinical testing [23] over the past decades, the number of effective therapies actually reaching routine practice has remained disappointingly low. To address this gap, several emerging avenues are being explored [110, 230]. These include the use of expanded chemical libraries—such as synthetic compounds or RNA-based molecules—as well as the application of high-throughput virtual screening and artificial intelligence to improve early candidate selection. In parallel, researchers are adopting new strategies to extract the maximum information from each experimental study and to minimize the well-known “translational bottleneck.” Innovations are also reshaping both ends of the pipeline: (a) on the preclinical side, human-based ex vivo cardiac models (e.g., organs-on-chips) may provide more predictive insights into treatment efficacy and safety; (b) on the clinical side, adaptive and smarter trial designs (e.g., integrating mechanism-, epidemiological-, *and* data-driven approaches) may offer the potential to accelerate evaluation while ensuring patient safety. Integrating these strategies can improve trial precision and translational relevance (Fig. 3). All together, these tools and approaches may help redefine the pathway from discovery to market, creating more efficient and effective therapies for cardiovascular disease, we hope.

## Data Availability

This is a review article, and therefore, there are no original data to be made available.

## Abbreviations

4-HNE: 4-Hydroxy-2-nonenal
AIF: Apoptosis-inducing factor
AKAP-121: A-kinase anchoring protein 121
Akt/PKB: Protein kinase B
AMI: Acute myocardial infarction
AMPK: AMP-activated protein kinase
ANT1: Adenine nucleotide translocator 1
CaMKII: Ca^2^^+^/calmodulin-dependent protein kinase II
CCR2: C–C chemokine receptor type 2
cGMP: Cyclic guanosine monophosphate
circRNA: Circular ribonucleic acid
DOCA: Deoxycorticosterone acetate
Dok-4: Docking protein 4
EMPA: Empagliflozin
ERK1/2,: Extracellular signal-regulated kinase 1/2
EVs: Extracellular vesicles
Fer-1: Ferrostatin-1
gp130: Glycoprotein 130
GPCR: G-protein coupled receptor
GSH: Glutathione
GSK-3β: Glycogen synthase kinase-3beta
HSPA12B: Heat shock protein A12B
IL-6: Interleukin-6
IL-37: Interleukin-37
lncRNA: Long non-coding RNAs
IPC: Ischemic preconditioning
IRI: Ischemia/reperfusion injury
JAK: Janus kinase
JNK: C-Jun N-terminal kinase
Lck: Lymphocyte-specific protein tyrosine kinase
MACE: Major adverse cardiovascular events
MEK: Mitogen-activated protein kinase kinase
MEKK1: Mitogen-activated protein kinase kinase kinase 1
Mfn1: Mitofusin-1
Mfn2: Mitofusin-2
MIF: Macrophage migration inhibitory factor
MLKL: Mixed lineage kinase domain-like protein
mPTP: Mitochondrial permeability transition pore
mtDNA: Mitochondrial DNA
NAC: *N*-Acetylcysteine
ncRNAs: Non-coding RNAs
NGF: Nerve growth factor
NLRP3: NLR family pyrin domain containing 3 protein
NO: Nitric oxide
NPs: Natriuretic peptides
Nrf2: Nuclear factor erythroid 2-related factor 2
p38: P38 mitogen-activated protein kinase
PAR: Poly(ADP-ribose)
PARP-1: Poly (ADP-ribosome) polymerase 1
PDE5: Phosphodiesterase type 5
pGC: Particulate guanylate cyclase
PI3K: Phosphatidylinositol-3-kinase
PINK1: PTEN-induced putative kinase 1
Pitx2: Pituitary homeobox 2
PKC-ε: Protein kinase C epsilon
PKG: Protein kinase G
PostC: Ischemic postconditioning
PRRs: Pattern recognition receptors
PTEN: Phosphatase and tensin homolog
RIC: Remote ischemic conditioning
RIPK1: Receptor-interacting protein kinase 1
RIPK3: Receptor-interacting protein kinase 3
RISK: Reperfusion injury salvage kinase
SAFE: Survivor activating factor enhancement
SFKs: Src-family kinases
sGC: Soluble guanylyl cyclase
SR: Sarcoplasmic reticulum
Src: Proto-oncogene tyrosine-protein kinase
SSO_2_: Supersaturated oxygen
STAT3: Signal transducer and activator of transcription 3
TAZ: Tafazzin
TEAD4: TEA domain transcription factor 4
TLR4: Toll-like receptor 4
TNF-α: Tumor necrosis factor α
TNF-R: Tumor necrosis factor receptor
VDAC: Voltage-dependent anion channel
YAP: Yes-associated protein
